# Case report: Image guidance retrieval of a foreign body in retropharyngeal space

**DOI:** 10.3389/fsurg.2022.949964

**Published:** 2022-07-27

**Authors:** Xingmei Wu, Yongquan Wang, Wei Sun, Xiaolin Zhu

**Affiliations:** Department of Otorhinolaryngology, The First Affiliated Hospital of Sun Yat-sen University, Guangzhou, China

**Keywords:** case report, foreign body, image guidance navigation, head and neck, retropharyngeal space

## Abstract

**Background:**

Ingested foreign bodies fully embedded in retropharyngeal space present a unique challenge, as they can be difficult to locate and visualize *via* classic transoral laryngoscopy or the transcervical approach.

**Methods:**

We retrieved a complete extraluminal chicken bone located in the patient's retropharyngeal space at the level of the C4-C5 spine through a well-designed transcervical approach with a combination of image-guided neck navigation.

**Conclusion:**

A combined use of image-guided neck navigation and a dedicated transcervical approach for location of a foreign body in the retropharyngeal space is practical and available for clinical application.

## Introduction

Foreign bodies fully embedded in retropharyngeal space, though uncommonly seen in clinical practice, present a unique challenge for surgeons. The difficulty of the retrieval surgery is mainly about how to precisely locate and visualize the foreign body. Certain techniques to reduce damage to surrounding structures and increase the success rate of operations are of crucial importance. Here, we reported a successful retrieval of a complete extraluminal foreign body located in the retropharyngeal space at the level of the C4-C5 spine through a well-designed transcervical approach with a combination of image guidance navigation. The study was conducted in accordance with ethical principles (Declaration of Helsinki), and informed consent was obtained from the patient.

**Figure 1:**
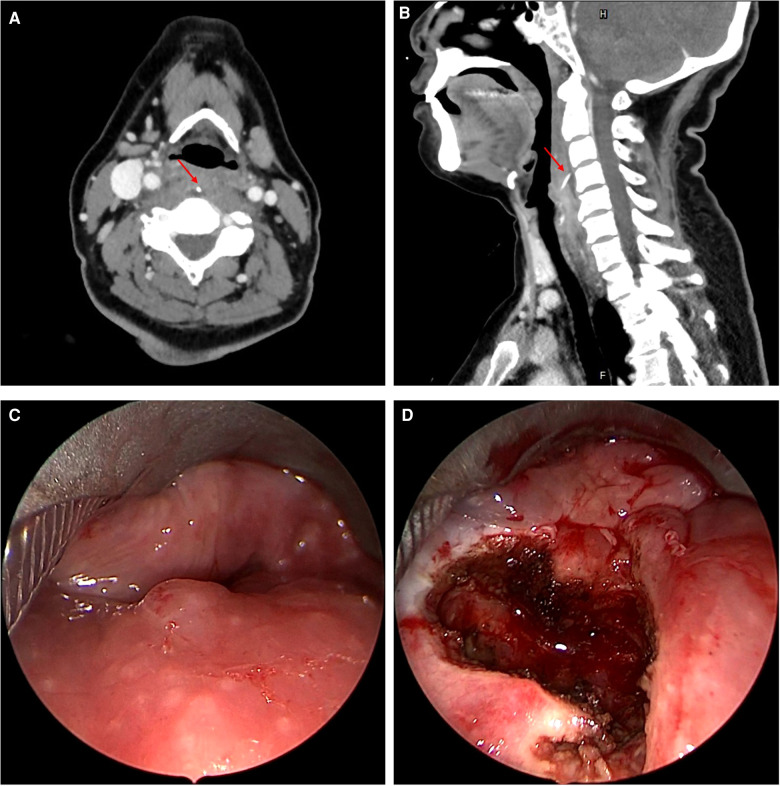
(**A**, **B**) Axial and sagittal CT images of patient on initial presentation; (**C**) laryngoscopy view of the posterior wall of the pharyngeal cavity before incision during the first surgery; (**D**) laryngoscopy view after incision of the pharyngeal mucosa during the first surgery. CT = computed tomography.

A 56-year-old woman presented with progressive odynophagia after swallowing a chicken bone six days earlier. Both flexible electronic laryngoscopy and upper gastrointestinal endoscopy failed to locate the chicken bone. Computed tomography (CT) revealed a 1.3 cm vertical shadow in the retropharyngeal space at the level of the C4 spine, with a distance of 0.8 cm toward the pharyngeal mucosa (Figures 1A, B). Next, a transoral rigid laryngoscopy was performed but ended up finding nothing after exploring the potential corresponding submucosal area of the posterior inferior pharyngeal wall.

**Figure 2:**
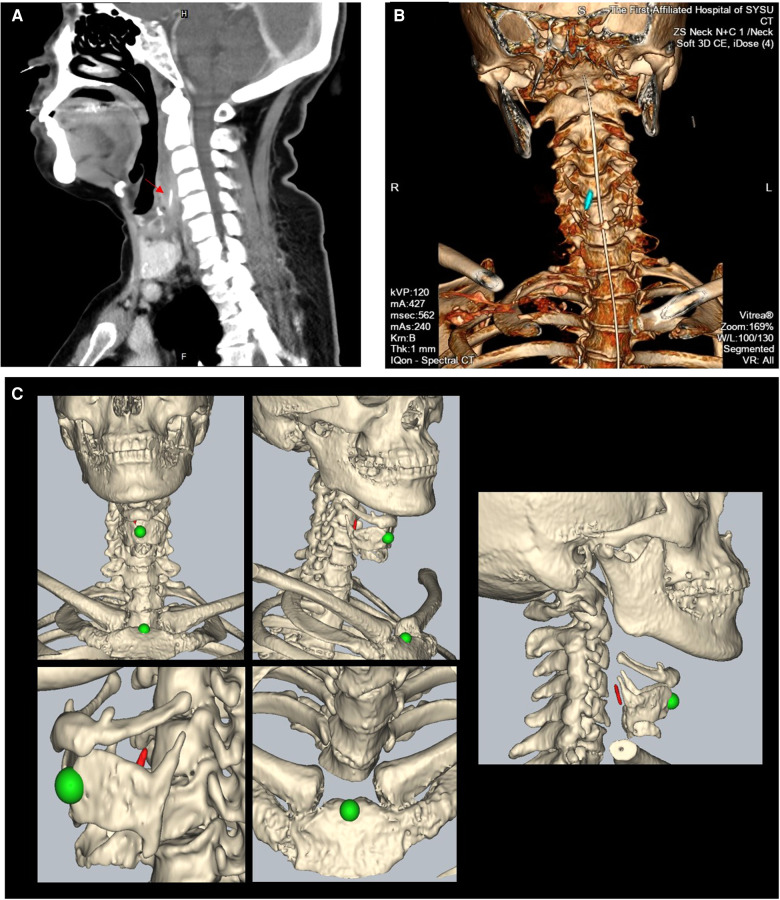
(**A**, **B**) Sagittal CT image and three-dimensional construction image of the patient after direct laryngoscopy ((**A**): the red arrow points to the foreign body); (**C**) representative images of intraoperative navigation registration: head and face contour scanning, combined with a facial marker, was used for registration, with an average registration accuracy not greater than 1.0 mm. After the model registration is completed, the patient's body position is further adjusted according to the positioning of the superior thyroid notch and the manubrium sterni (green) in the navigation model, so that the neck position from the navigation model matches the actual position of the patient’s neck.

**Figure 3:**
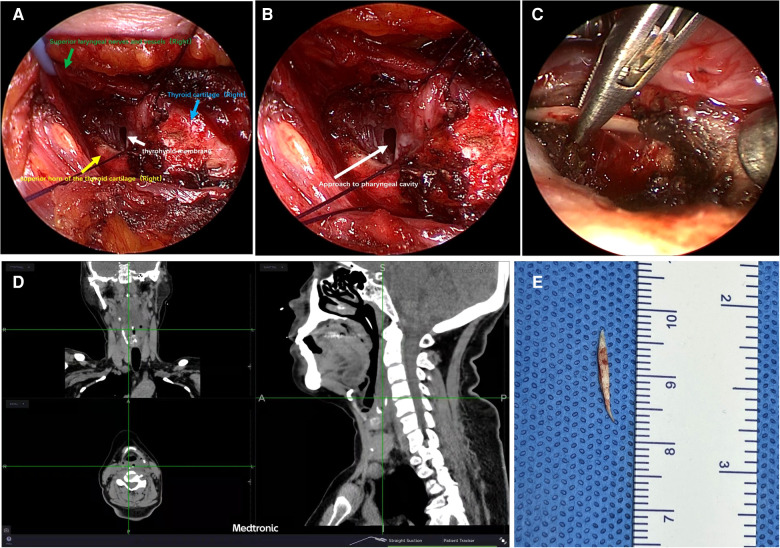
Representative images of transcervical surgery with the combined use of both endoscope and image guidance navigation. (**A**) Exposure of the thyroid cartilage, thyrohyoid membrane, superior horn of the thyroid cartilage, and superior laryngeal nerves; (**B**) incision of a thyrohyoid membrane involved making an opening to the pharyngeal cavity; (**C**) successful location and retrieval of a foreign body from retropharyngeal space; (**D**) intraoperative positioning of the foreign body using image guidance navigation; (**E**) image of the foreign body.

Experience from the unsuccessful attempts to locate the foreign body indicated a need to reconsider the operative strategy. Image guidance was proposed and adopted to help solve the issue. Thus, the neck CT scan was repeated with a head navigation CT scan three days after the first surgery. As shown in Figure 2, the chicken bone was retained but displaced downward to the C4-C5 spine. The second surgical attempt for retrieval was implemented one day later. On the basis that image-guided navigation will be interfered under rigid laryngoscopy, we employed a transcervical approach to enter the pharyngeal cavity with minimum damage to surrounding structures. During this surgery, a 5 cm incision was made over the left upper neck. Incision of the thyrohyoid membrane from the front of the right superior horn of the thyroid cartilage was performed to expose the pharyngeal cavity. The corresponding internal laryngeal nerve and the superior laryngeal artery were well recognized and protected in time (Figures 3A, B). Medtronic intraoperative image guidance navigation was used to locate the pharyngeal mucosa incision position for foreign body visualization (Figure 3D). Taking the mobility of the soft tissues of the neck related to the cartilages and bony structures into consideration, we ensured that the position of the head and neck during surgery was in accordance with its position when the CT scan was obtained. A 1.3 cm chicken bone was successfully found beneath the incised pharyngeal mucosa and removed *en bloc* without any injury to the internal laryngeal nerves and thyroid cartilage (Figures 3C, E). The successful operation turned out to be a pleasant surprise to the patient and her family members who had thought it might be a failure again. The patient then received enteral nutrition *via* the nasal gastric tube for two weeks. Telephone follow-up showed satisfactory treatment effect without severe complications.

## Description of the neck navigation technique

1. Preoperative navigation CT scanning preparation: The CT scanning range was from the top of the head to the manubrium sterni, with a slice thickness of 1 mm. The scanning position of the patient was the same as that of the operation.
2. Intraoperative navigation registration: Use head and face contour scanning, combined with a facial marker for registration, with an average registration accuracy not greater than 1.0 mm. After the model registration is completed, the patient's body position is further adjusted according to the positioning of the superior thyroid notch and the manubrium sterni in the navigation model, so that the neck position from the navigation model matches the actual position of the patient's neck.
3. Intraoperative manipulation: Connect and register the appliance for navigation, and use the superior thyroid notch and the superior horn of the thyroid cartilage to check the navigation accuracy. If there is an error, adjust the position of the neck to achieve coherence. After the verification, you can use the navigation appliance to locate the position of the foreign body.

## Specific perioperative considerations

In our preoperative preparation, the feasibility of transoral laryngoscopy with image guidance was proposed but precluded due to the following reasons: first, the foreign body was located at the C4-C5 spine level that required the rigid metal laryngoscope to achieve transoral exposure, which would lead to an invalidation of image guidance; second, compared with the preoperative CT position, the relative position between head and neck changes under transoral laryngoscopy, which would result in neck navigation inaccuracy and eventually failure of foreign body location.

## Discussion

To the best of our knowledge, this is the first report of image-guided migrated ingested foreign body retrieval in the neck. Based on conventional navigation practice, we obtained both head and neck static CT images preoperatively and thus extended the navigation scope from the head toward the neck during an image-guided exploratory attempt.

Retrieval of extraluminal ingested foreign bodies in the neck could be of great challenge for clinicians while using classic surgical approaches. Currently, there are no published clinical guidelines or expert consensus discussing the retrieval of foreign bodies from deep neck structures. Adjuvant navigation means or a combination of multiple surgery approaches might help increase the success rate of locating foreign bodies (1). Both intraoperative ultrasound guidance and image guidance have been considered as minimally invasive and readily available implements based on a few studies and reports (2). While ultrasound guidance seeks talented surgeons or assistance from professional judgment, image guidance is more extensively developed and managed by otorhinolaryngologists. However, most published data showed the application of image-guided navigation surgeries confined to the head (3). Devaraja and co-authors (4) described a “minimally invasive approach” for the retrieval of a retropharyngeal foreign body through the use of an endoscope *via* a transcervical approach without entering the pharyngeal cavity, which required an excellent surgical technique and great confidence. Instead, we here introduced a concise and repeatable technique to enter the pharyngeal cavity without injury to the superior laryngeal nerves and thyroid cartilage. Similarly, we also used an endoscope for surgical vision lightning of the pharyngeal cavity during the transcervical operation. Such a cervical approach has both advantages and limitations, as it provides safer operational vision at the expense of inevitably increasing surgical wounds. In general, we consider such operative strategy a tactful reference to help relieve surgeon stress and improve satisfaction levels.

## Conclusion

A combined use of image-guided neck navigation and a dedicated transcervical approach for location of a foreign body in retropharyngeal space is practical and available for clinical application.

## Data Availability

The original contributions presented in the study are included in the article/Supplementary Material; further inquiries can be directed to the corresponding author/s.
